# quickBAM: a parallelized BAM file access API for high-throughput sequence analysis informatics

**DOI:** 10.1093/bioinformatics/btad463

**Published:** 2023-07-27

**Authors:** Anders Pitman, Xiaomeng Huang, Gabor T Marth, Yi Qiao

**Affiliations:** UTAH Center for Genetic Discovery, Department of Human Genetics, University of Utah School of Medicine, 15 N 2030 E, Salt Lake City, UT 84112, United States; UTAH Center for Genetic Discovery, Department of Human Genetics, University of Utah School of Medicine, 15 N 2030 E, Salt Lake City, UT 84112, United States; UTAH Center for Genetic Discovery, Department of Human Genetics, University of Utah School of Medicine, 15 N 2030 E, Salt Lake City, UT 84112, United States; UTAH Center for Genetic Discovery, Department of Human Genetics, University of Utah School of Medicine, 15 N 2030 E, Salt Lake City, UT 84112, United States

## Abstract

**Motivation:**

In time-critical clinical settings, such as precision medicine, genomic data needs to be processed as fast as possible to arrive at data-informed treatment decisions in a timely fashion. While sequencing throughput has dramatically increased over the past decade, bioinformatics analysis throughput has not been able to keep up with the pace of computer hardware improvement, and consequently has now turned into the primary bottleneck. Modern computer hardware today is capable of much higher performance than current genomic informatics algorithms can typically utilize, therefore presenting opportunities for significant improvement of performance. Accessing the raw sequencing data from BAM files, e.g. is a necessary and time-consuming step in nearly all sequence analysis tools, however existing programming libraries for BAM access do not take full advantage of the parallel input/output capabilities of storage devices.

**Results:**

In an effort to stimulate the development of a new generation of faster sequence analysis tools, we developed quickBAM, a software library to accelerate sequencing data access by exploiting the parallelism in commodity storage hardware currently widely available. We demonstrate that analysis software ported to quickBAM consistently outperforms their current versions, in some cases finishing an analysis in under 3 min while the original version took 1.5 h, using the same storage solution.

**Availability and implementation:**

Open source and freely available at https://gitlab.com/yiq/quickbam/, we envision that quickBAM will enable a new generation of high-performance informatics tools, either directly boosting their performance if they are currently data-access bottlenecked, or allow data-access to keep up with further optimizations in algorithms and compute techniques.

## 1 Introduction

High-throughput, genome wide next-generation sequencing (NGS) has revolutionized precision medicine. As an example, NGS has now been implemented as a routine diagnostic modality in many pediatric subspecialty clinics for critically ill children admitted into the neonatal intensive care unit or pediatric intensive care unit (Petrikin *et al.* 2015, Elliott *et al.* 2019). And increasingly, genomics-guided precision medicine is helping advanced cancer patients who have exhausted standard-of-care options (Schwartzberg *et al.* 2017). In these settings, the amount of data analyzed is small compared to large cohort studies, involving usually one to a few tumor samples and a paired normal sample from the same patient. However, fast analysis turnaround is of critical importance. Furthermore, after the optimal treatment is identified, it still takes significant time to coordinate treatment access due to e.g. drug acquisition, compassionate care approval, clinical trial enrollment, or insurance authorization. It is therefore significant that the informatics analysis tasks, which have surpassed sequencing as the primary bottleneck, are to be as fast as current computer hardware can make possible.

The BAM file format (Li *et al.* 2009) is the current *de facto* standard for storing sequencing data generated from NGS experiments. BAM files are the most common starting place for various downstream analyses. The BAM format is the compressed, binary version of the SAM format, which we designed as part of the 1000 Genomes Project (1000 Genomes Project Consortium *et al.* 2015) to reconcile the once many different formats of storing sequencing data. Subsequently, software libraries are created to provide APIs to access the sequencing reads contained in a BAM file. HTSLIB (Bonfield *et al.* 2021) is the file access layer from Samtools (Li *et al.* 2009), the software developed to perform many SAM/BAM file related operations. The main focus of HTSLIB is to provide high level abstractions so that the programming interfaces stay the same regardless of the underlying file format (be it SAM, BAM, or CRAM) or storage and transport media (local files, HTTP URLs, or cloud storage). BamTools (Barnett *et al.* 2011) and SeqLib (Wala and Beroukhim 2017) focus on modern C++ API designs for ease of programming. While BamTools implements its own BAM parsing logic, SeqLib integrates HTSLIB as the access layer. These libraries perform file access in a single-threaded manner (HTSLIB does support multi-threaded decompression, but not file reading), which is a reasonable design choice when (i) informatics analysis is compute bottlenecked, and therefore cannot benefit from faster data access; or (ii) the file storage and transport media are incapable of high performance, e.g. when the files are served over low-bandwidth network attached storages. However, these tools cannot take advantage of storage technologies that are capable of much higher levels of file I/O parallelization and data bus bandwidth.

Specifically, we are concerned with two generally available storage technologies. On premise, the Lustre distributed file system is capable of achieving very high aggregated bandwidth by striping files onto different computer nodes and hard drives. And on the cloud such as the commercial Amazon Web Services (AWS), it is already commonplace to instantiate nonvolatile memory express solid state drives (NVMe SSDs) as the primary storage media. These two types of storage solutions cover the majority of high-performance computing facilities, and both provide enough parallelism to support much higher analysis throughput than currently utilized.

## 2 Materials and methods

**Figure 1.**
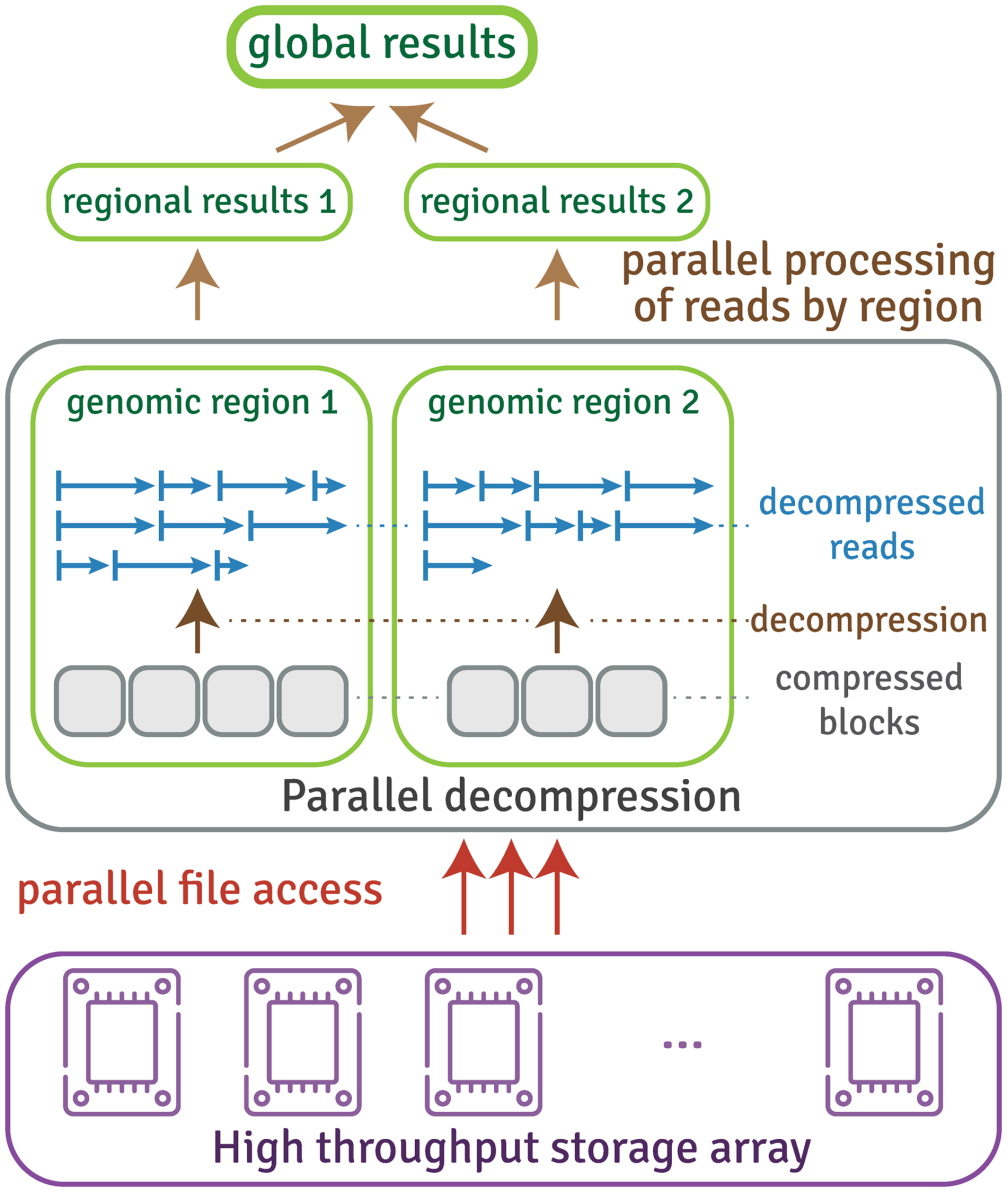
Parallel processing architecture using quickBAM. quickBAM utilizes the scatter/gather paradigm to parallelize data access and computation tasks across many genomic regions before combining the regional results to produce global results.

We developed quickBAM (Fig. 1), which uses two strategies to parallelize BAM file access. First, when the bam file index (BAI) is available, we utilize the “fixed-bin” indices which contain the starting file offset of each 16-kb genomic window. Second, when the BAI is not available (unsorted/unindexed BAMs or the unmapped region in indexed BAMs), we use a heuristic scanner (see Supplementary Methods) to directly locate multiple starting locations for parallel parsing. Since the majority of sequence analysis tasks (e.g. quality control, various types of mutation calling) involve reading BAM files, quickBAM has the potential to significantly shorten end-to-end analysis turnaround. quickBAM is freely available at https://gitlab.com/yiq/quickbam/ with extensive accompanying documentation available at https://yiq.gitlab.io/quickbam/.

## 3 Results

### 3.1 Benchmarking of operating system I/O APIs

**Figure 2.**
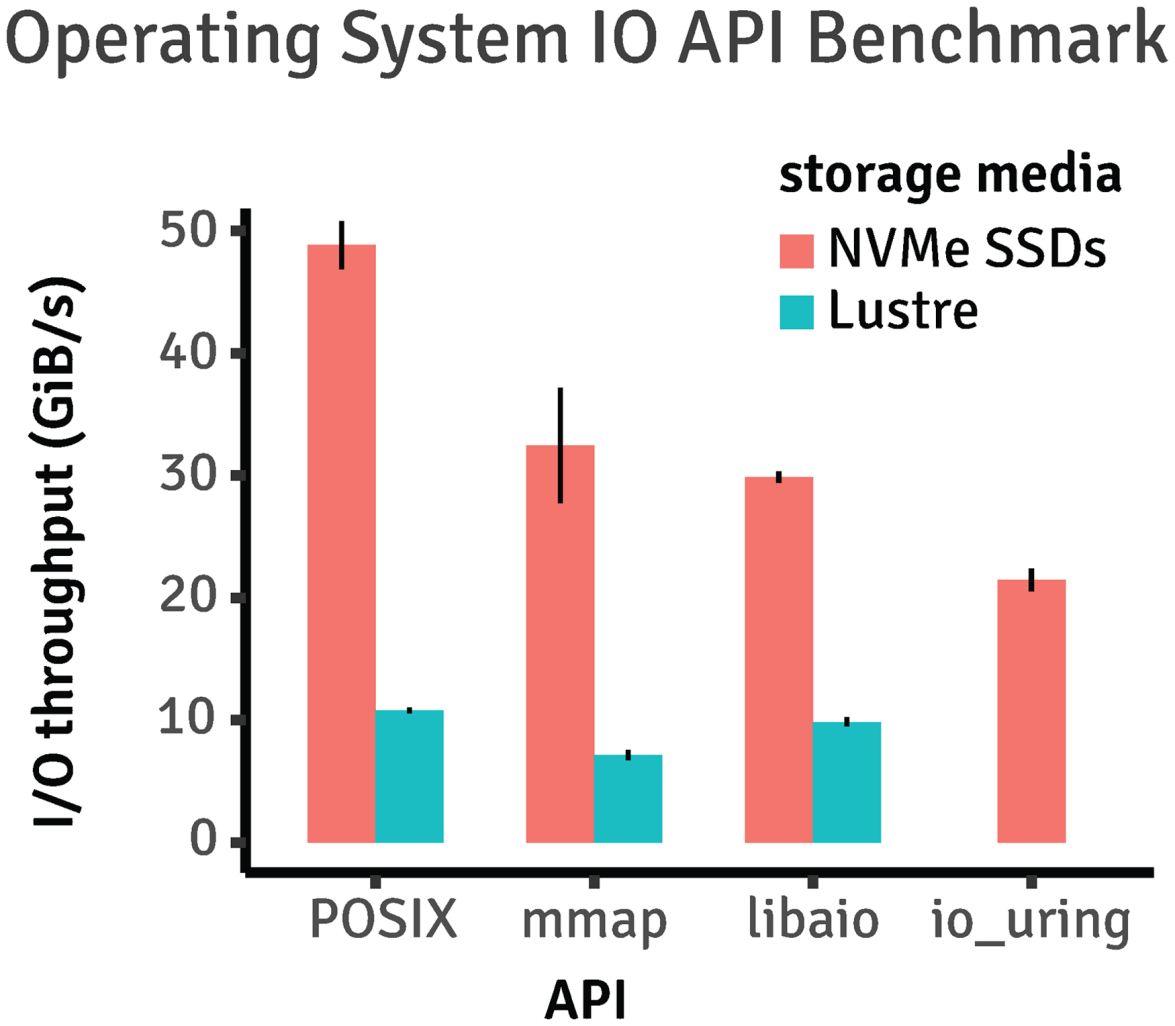
Benchmarking results of various operating system IO APIs. Sequential reads of 4k blocks were carried out using POSIX, memory map (mmap), and libaio on both NVME SSD available on AWS and a Lustre distributed file system available to our local compute cluster. We in addition benchmarked *io_uring* but only on AWS as it is supported by only recent Linux kernels not yet available at our local facility.

Since our work is focused on extracting the maximum I/O performance, we start with benchmarking various operating system I/O APIs. While the POSIX synchronous I/O APIs like *fread()* have been the long-standing standard, newer APIs such as *libaio* and *io_uring* now exist with promises to deliver better performance. Using the *fio* benchmarking utility, we carried out parallel read bandwidth benchmarking on both a Lustre distributed system available locally in our facility, as well as a 4-way NVMe SSD raid0 array available via AWS. We evaluated four APIs on AWS: POSIX synchronous, memory map, *libaio*, and *io_uring*; while dropping *io_uring* on our local Lustre because it is not yet supported by the Linux kernel deployed at our high-performance compute cluster. As shown in Fig. 2 (raw data available in Supplementary Table S1), the POSIX synchronous APIs consistently outperform other APIs on both Lustre and SSD arrays. Therefore, we chose to use POSIX synchronous APIs for our work.

### 3.2 Performance evaluation strategies

In the next two sections, we describe performance improvements with example algorithms reimplemented using quickBAM. Briefly we describe the benchmarking strategies here with details available in Supplementary Methods. For each algorithm, we benchmarked its performance using two whole genome datasets: Genome In A Bottle (Zook *et al.* 2016) Illumina 2x250 bam files (HG002 and HG004) with a nominal coverage of 75X; and a tumor normal pair 60x Illumina sequencing bam files (Bn2 and Germ1) from a published study (Huang *et al.* 2021). The former dataset is to facilitate result reproduction since it is openly accessible; whereas the latter is to provide a more appropriate tumor context, whose genomes can be highly aberrant. The same tests are carried out separately on Lustre storage at our local cluster and NVMe SSDs on AWS. There are 80 and 96 hyper-threaded cores on our local and AWS servers respectively. Therefore, our Lustre benchmarks contain run configurations of 80, 60, 40, 20, 10, and 1 threads; and NVMe SSD benchmarks contain 96, 72, 48, 24, 12, and 1 threads. For each test, an effective throughput is calculated as total input file size divided by measured total time-till-completion. Each test is repeated three times to account for uncontrollable variabilities.

### 3.3 Proof-of-concept implementation of samtools flagstats and performance evaluation

**Figure 3.**
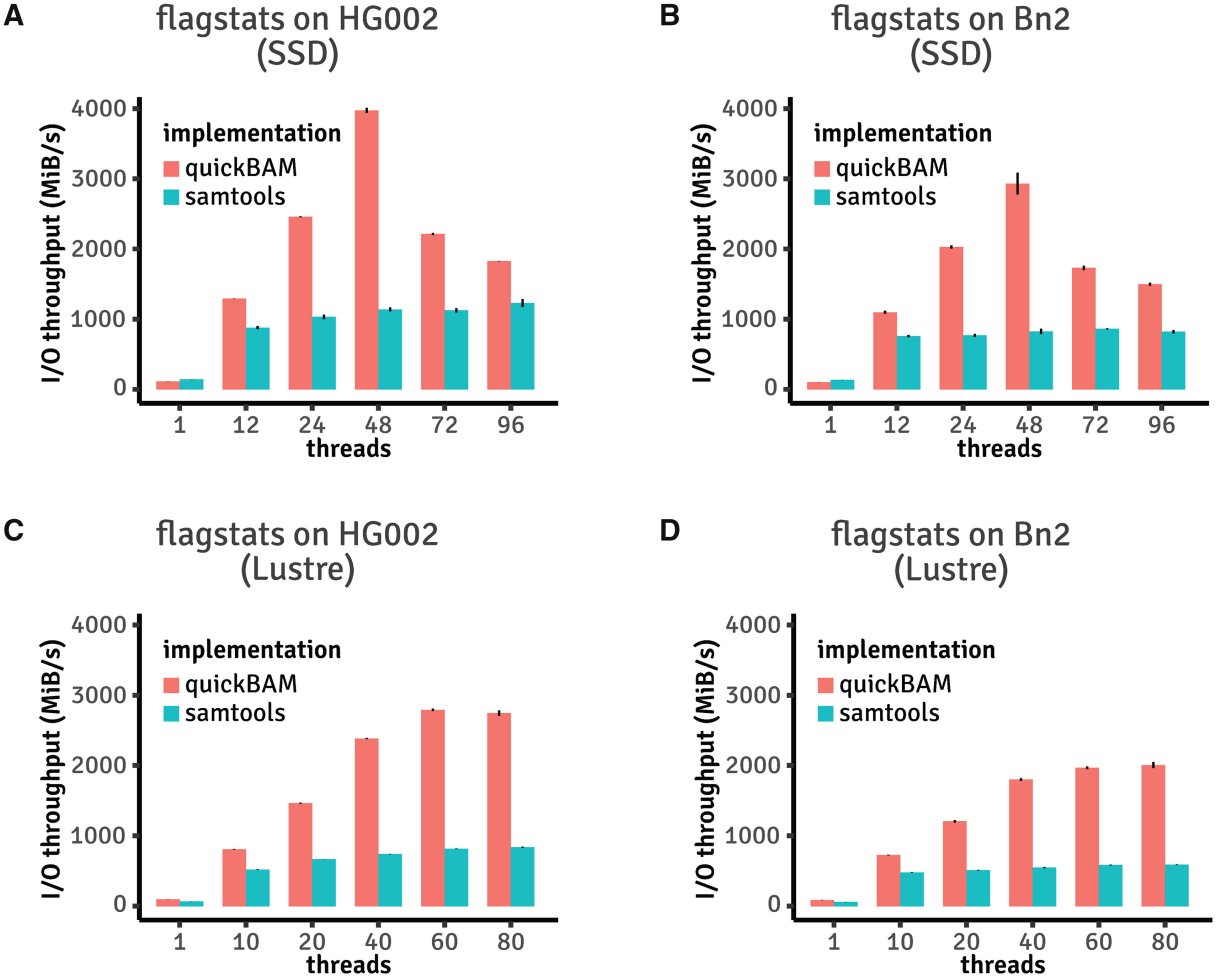
Performance benchmark results of reimplemented flagstats versus stock version. (A) Results using the GIAB HG002 Illumina 2x250 BAM file with an NVMe SSD array available on AWS. (B) Results using the rapid autopsy Bn2 sample BAM file with an NVMe SSD array available on AWS. (C) Results using the GIAB HG002 Illumina 2x250 BAM file with a Lustre distributed file system. (D) Results using the rapid autopsy Bn2 sample BAM file with a Lustre distributed file system.

The first sample program we ported to quickBAM is the utility in samtools called flagstats. Flagstats iterates over the entire BAM file, updating statistics (e.g. number of reads failed QC) with the flags field of each read, and finally printing the statistics when all reads are processed. It is a simple algorithm, however it serves the purpose of demonstrating performance gain via parallelization. Using quickBAM, it is possible to compute separate statistics for each 16-kb window across the entire genome. This 16-kb window is chosen because it directly maps to the linear indices in the bam index file. Reads that overlap window boundaries are partitioned into the earlier window to avoid double counting. Since the data structure of flagstats consists of only integer counters, the “gather” stage is a simple summation of these counters from all windows. With a single thread, quickBAM based flagstats and samtools show similar performances (Fig. 3). However, while samtools benefits little from more than 10 threads, quickBAM allows for a much better scaling. Full timing observations are listed in Supplementary Table S2. The quickBAM version of flagstats produces identical results compared to the samtools version. Other algorithms that can potentially be implemented in a similar fashion include, but are not limited to, read counting per fixed genome windows for CNV detection and transcript abundance counting per gene in RNAseq data analysis.

### 3.4 Reimplementation of a real-world, widely used program and performance evaluation

**Figure 4.**
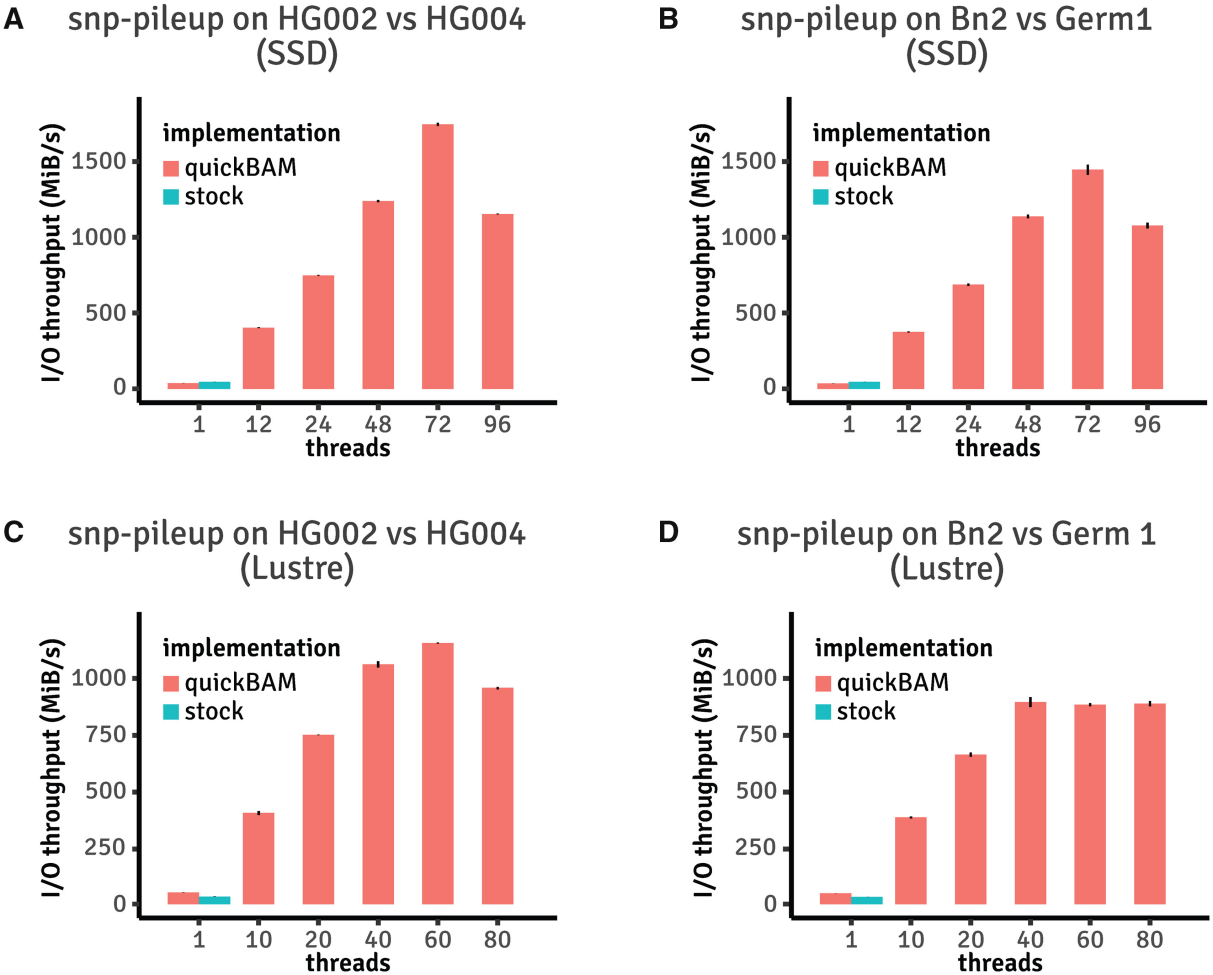
Performance benchmark results of reimplemented *snp-pileup* versus stock version. Note that the stock implementation of *snp-pileup* does not support multi-threading. (A) Results using the GIAB HG002 and HG004 Illumina 2x250 BAM files with an NVMe SSD array available on AWS. (B) Results using the rapid autopsy Bn2 and Germ1 BAM files with an NVMe SSD array available on AWS. (C) Results using the GIAB HG002 and HG004 Illumina 2x250 BAM file with a Lustre distributed file system. (D) Results using the rapid autopsy Bn2 and Germ1 BAM file with a Lustre distributed file system.

The second sample program we ported to quickBAM is a utility found in the somatic copy number variant detection algorithm FACETS (Shen and Seshan 2016) called “*snp-pileup*.” *Snp-pileup* takes as input a set of BAM files and a VCF file [commonly the dbSNP published human common polymorphic sites (Sherry *et al.* 1999)], and iterates over positions in the VCF file. At each position, it pulls all the reads from the BAM files overlapping with the position, and extracts the sequencing coverage and variant allele fraction information. Different from the *flagstats* example which parallelizes over multiple, nonoverlapping genomic windows, we ported *snp-pileup* to parallelize over groups of consecutive variant positions. As shown in Fig. 4, quickBAM *snp-pileup* achieved over 1.5 GiB/s data processing throughput with quickBAM’s built-in multiple input pileup engine, more than 38 times faster than the original implementation which does not support multithreading (HG002 & HG004 on AWS, 1744.51 MiB/s quickBAM versus 46.38 MiB/s stock). Consequently, using the Genome In A Bottle (Zook *et al.* 2016) HG002 and HG004 Illumina 2x250bp BAM files (242 GiB of data combined), a two samples joint *snp-pileup* can be finished in 2 min 20 s (quickBAM), compared to 1 h 29 min (original version). A similar speedup is observed with the tumor normal dataset. Full timing observations are listed in Supplementary Table S3. We note that the quickBAM version of *snp-pileup* produces nearly identical results compared to the original version, differing at 217 out of 28.6 million positions (GIAB) and 1879 out of 28.2 million positions (tumor normal). We discuss this in detail below. Other algorithms that can potentially be implemented in a similar fashion include single cell sequencing data genotyping and variant calling.

We traced the small differences between the original and quickBAM *snp-pileup* to three types of edge cases. The first, which accounts for 10% of the differences, is due to a bug in the stock *snp-pileup* program that would erroneously skip over a location when the input VCF file contains duplicate genomic coordinates, and the first occurrence is not a bi-allelic SNP site. The second, which accounts for 75% of the differences, is due to the difference between how the multiple pileup engines in HTSLIB and quickBAM enforce limits in extremely high coverage regions. HTSLIB uses a “pileup iterator” to which reads are added until the limit is reached, and no more reads will be added until one is removed from the iterator. The pileup engine in quickBAM, however, tries to use every read to update the pileup information at each genomic location. The limit is built into the per-location data structure instead. This generally results in quickBAM counting more even coverage. If there is a demand from the community, we will update the quickBAM multiple pileup engine to behave exactly as HTSLIB in high coverage regions. For the third type of edge case (15% of the differences), we verified that quickBAM produced the same results as a third pileup program: *samtools mpileup*. Since the stock *snp-pileup* uses the same HTSLIB multiple pileup engine as *samtools mpileup* does, the exact reason for these differences cannot be determined without putting significant debugging efforts into the original *snp-pileup* program. We thus conclude that these differences are unlikely due to mistakes in quickBAM.

## 4 Discussion

In this manuscript, we present quickBAM, a software library for accessing sequence alignments in BAM files with a high degree of parallelization and performance. We achieve this by taking advantage of parallel file access supported by modern storage hardware. As a demonstration of quickBAM’s utility, we ported various types of sequence analysis algorithms, which have shown consistently higher performance than their original implementations. Performance gain is observed with both common on-premise storage solutions such as Lustre, and with new storage hardware such as NVMe SSDs available commonly on the cloud. Porting algorithms to quickBAM offers significant analysis time reduction. As demonstrated by the *snp-pileup* benchmark results, a tumor-normal pair 60X WGS dataset, which took 1.5 h to process using the original version, can be finished in just under 2.5 min with a quickBAM implementation on the same hardware. While not being the primary focus of this manuscript, we further benchmarked quickBAM’s performance on slow storage media (Supplementary Table S4). While quickBAM and samtools can both easily saturate the storage bandwidth with a moderate number of threads, quickBAM showed a performance advantage with as few as two threads.

Interestingly, our results show that, while quickBAM allows much higher performance scalability with respect to increasing parallelism, the performance eventually starts to decline as the number of threads approaches the total number of hyperthreaded cores (with the exception of flagstats on Lustre). This is likely due to oversubscribing system resources, and resulting in software and hardware scheduling overhead exceeding the benefit of increased parallelism. Therefore, it suggests that “sweet-spots” exist for specific hardware/software combinations, and should be determined with trial runs. We should point out that our benchmarking results are focused on high-quality human sequencing data. In other situations, such as highly fragmented plant genomes or low mean coverage sequencing technologies like ChIP-seq, the observed performance gain may or may not generalize. Special care on how work tasks are partitioned are likely needed to maximize parallel efficiency.

Our framework encourages “internal parallelism” i.e. one copy of the analysis program is launched which performs job division and coordinates multi-threading, as opposed to “external parallelism” i.e. many copies of the same analysis programs are launched with each one configured to perform a subset of the total work. There are two benefits of internal parallelism we recognize. First, it is generally easier to program the job division/results gathering processes within the same program space as the actual work routines. We took advantage of this in *snp-pileup* to partition jobs roughly according to the size of data each job spans using the BAM index. And second, since the number of jobs created is independent of the number of threads (with the help of thread pools), it is easier to avoid hardware oversubscription while at the same time benefit from load balancing via job stealing i.e. an idling thread can take jobs from a busy thread to maximize hardware utilization. The trade-off, however, is that internal parallel programs are great at scaling up, but not at scaling out. In future work, we plan to incorporate external parallelism mechanisms such as the OpenMPI software library (Gabriel *et al.* 2004) to make quickBAM even more scalable. Other features planned for future updates after our initial release are CRAM support and remote URL access support.

Our work enables many types of sequence analysis software to be accelerated significantly, which in turn benefit time sensitive clinical/research applications such as precision medicine. Our code is open source and publicly available with extensive documentation and sample programs. We plan to actively maintain the project, incorporating further improvements and developing new features according to feedback from the user community.

## Supplementary Material

btad463_Supplementary_Data

## Data Availability

GIAB Ashkenazim Trio HG002 and HG004 Illumina 2x250bp novoalign GRCh38 BAM files are available at https://ftp-trace.ncbi.nlm.nih.gov/ReferenceSamples/giab/data/AshkenazimTrio/HG002_NA24385_son/NIST_Illumina_2x250bps/novoalign_bams/. https://ftp-trace.ncbi.nlm.nih.gov/ReferenceSamples/giab/data/AshkenazimTrio/HG004_NA24143_mother/NIST_Illumina_2x250bps/novoalign_bams/. The rapid autopsy tumor normal sample dataset was from a published study (Huang et al., 2021). Known polymorphism sites VCF used for the snp-pileup benchmark experiments are available at ftp://ftp.ncbi.nlm.nih.gov/snp/organisms/human_9606/VCF/00-common_all.vcf.gz.

## References

[btad463-B1] 1000 Genomes Project Consortium. A global reference for human genetic variation. Nature 2015;526:68–74. 26432245 10.1038/nature15393 PMC4750478

[btad463-B2] Barnett DW , Garrison EK, Quinlan AR et al BamTools: a C++ API and toolkit for analyzing and managing BAM files. Bioinformatics 2011;27:1691–2. 21493652 10.1093/bioinformatics/btr174 PMC3106182

[btad463-B3] Bonfield JK , Marshall J, Danecek P et al HTSlib: C library for reading/writing high-throughput sequencing data. Gigascience 2021;10:giab007. 33594436 10.1093/gigascience/giab007 PMC7931820

[btad463-B4] Elliott AM , Du Souich C, Lehman A et al RAPIDOMICS: rapid genome-wide sequencing in a neonatal intensive care unit-successes and challenges. Eur J Pediatr 2019;178:1207–18. 31172278 10.1007/s00431-019-03399-4

[btad463-B5] Gabriel E , Fagg GE, Bosilca G et al Open MPI: goals, concept, and design of a next generation MPI implementation. In: Kranzlmüller D, Kacsuk P. and Dongarra J (eds) Recent Advances in Parallel Virtual Machine and Message Passing Interface. Berlin, Heidelberg: Springer, 2004, 97–104.

[btad463-B6] Huang X , Qiao Y, Brady SW et al Novel temporal and spatial patterns of metastatic colonization from breast cancer rapid-autopsy tumor biopsies. Genome Med 2021;13:170. 34711268 10.1186/s13073-021-00989-6 PMC8555066

[btad463-B7] Li H , Handsaker B, Wysoker A et al; 1000 Genome Project Data Processing Subgroup. The sequence alignment/map format and SAMtools. Bioinformatics 2009;25:2078–9. 19505943 10.1093/bioinformatics/btp352 PMC2723002

[btad463-B8] Petrikin JE , Willig LK, Smith LD et al Rapid whole genome sequencing and precision neonatology. Semin Perinatol 2015;39:623–31. 26521050 10.1053/j.semperi.2015.09.009 PMC4657860

[btad463-B9] Schwartzberg L , Kim ES, Liu D et al Precision oncology: who, how, what, when, and when not? Am Soc Clin Oncol Educ Book 2017;37:160–9. 28561651 10.1200/EDBK_174176

[btad463-B10] Shen R , Seshan VE. FACETS: allele-specific copy number and clonal heterogeneity analysis tool for high-throughput DNA sequencing. Nucleic Acids Res 2016;44:e131. 27270079 10.1093/nar/gkw520 PMC5027494

[btad463-B11] Sherry ST , Ward M, Sirotkin K et al dbSNP-database for single nucleotide polymorphisms and other classes of minor genetic variation. Genome Res 1999;9:677–9. 10447503

[btad463-B12] Wala J , Beroukhim R. SeqLib: a C ++ API for rapid BAM manipulation, sequence alignment and sequence assembly. Bioinformatics 2017;33:751–3. 28011768 10.1093/bioinformatics/btw741 PMC5859992

[btad463-B13] Zook JM , Catoe D, McDaniel J et al Extensive sequencing of seven human genomes to characterize benchmark reference materials. Sci Data 2016;3:160025. 27271295 10.1038/sdata.2016.25 PMC4896128

