# Supplementary material for: quickBAM: a parallelized BAM file access API for high-throughput sequence analysis informatics
Source: Bioinformatics. 2023 Jul 27;39(8):btad463. doi: 10.1093/bioinformatics/btad463 (PMC10412403; doi:10.1093/bioinformatics/btad463)
Supplement: btad463_Supplementary_Data [file btad463_supplementary_data.zip › SuppMethods.docx]

**SUPPLEMENTAL METHODS**

1. Core API design principles.

We designed our API according to the following principles.

1. The library interface is in C-style data structures and functions that operate on these data structures.
2. To avoid costly abstractions, the data structures are implemented as C structs. The struct members are aligned to the byte arrangement of the records in a BAM or BAI file they represent. The members are intended to be accessed by client programs directly.
3. Functions that perform operations on the data structures are named as <struct_name>_<operation_name> for better code readability.
4. Modern C++11 and C++14 features are used whenever it makes sense. This includes
   1. Smart pointers are used for automatic memory management
   2. Iterator types in quickBAM often support the ***range-for*** syntax
   3. STL algorithms are preferred over explicit loops
   4. Auto type deduction is used whenever possible
5. The implementation of quickBAM uses openMP and Intel Thread Building Block (libtbb) for parallelization.

2. BAM file partitioning algorithm

QuickBAM speeds up sequence analysis through parallelizing data access and computation over many smaller regions across the genome, which translates to different parts of a BAM file. When the BAM index is available, this is straightforward: spawn a separate task for each 16kb genomic window. This is because BAI directly records the file offset where parsing should start for each 16kb genomic window across the entire genome. Note that 16kb offers the finest level of parallelism. Client code can choose to combine multiple such windows to reduce the number of separate tasks generated. Here we describe in detail when BAI is not available, such as in the case of unsorted BAM files, or the unmapped reads region in an indexed BAM file.

The data stored in a BAM file consists of a series of BGZF compression blocks. A BGZF block contains information about how large the block is. Therefore, it represents a singly linked list data structure i.e. if the location of one BGZF block is already known (e.g. the first one in a BAM file), the location of the next BGZF block can be calculated by *thisBlock.offset + thisBlock.block_size*. However, when the offset of a BGZF block is not known (e.g. attempting to start reading a BAM file somewhere in the middle), the data structure does not directly indicate where the next BGZF block starts. We use a heuristic algorithm to scan for the next BGZF block using four magic numbers that are guaranteed by the BAM file specification to be present in a BAM file BGZF block: 31(byte 0), 139(byte 1), 66(byte 12), and 67(byte 13). This allows us to scan a BAM file from multiple arbitrary offsets, and find the next valid BGZF blocks. Once a valid BGZF block is located, the rest can be located using *block_size* values.

The bigger challenge of this approach lies in the data stream after decompressing a series of BGZF blocks. That data stream contains the BAM alignment records that do not necessarily align with the beginning of a BGZF block. Since BAM records are similarly a singly linked list, we need to scan the decompressed data to locate the first valid BAM record. This is more difficult because BAM records do not have predictable magic numbers. We instead use properties of a BAM record to heuristically verify if we have found a valid one. We currently implement the following criteria:

- -1 <= refID < n_ref (n_ref is the number of references, part of the BAM header)
- pos == 0 if refID == -1; else pos < l_ref[refID] (l_ref is the lengths of references, part of BAM header)
- -1 <= next_refID < n_ref
- next_pos == 0 if next_refID == -1; else next_pos < l_ref[next_refID]
- read_name[l_read_name - 1] == 0 (this is because read name is null-terminated)

This set of criteria is derived from the definitions of these properties, and should be agnostic to sequencing technologies / read length / species. Using the datasets mentioned in this manuscript, we have not yet encountered a false positive case. We will optimize our criteria list in future updates if false positive cases are discovered.

With both of these scanning techniques, we are able to parallelize data access across an un-indexed BAM file, or the unmapped reads in an indexed BAM file into which the index does not provide finer partitioning. We note that this approach only applies to read-based algorithms (e.g. flagstats) as position-based algorithms (e.g. snp-pileup) by definition only operates on coordinate-sorted BAM files in mapped regions.

3. Benchmark experiments environment

In this section we describe the compute environment setup for our benchmark experiments to facilitate results reproduction.

3.1. Benchmarking using SSD storage array on AWS

We performed benchmark experiments on the Amazon Web Service (AWS) cloud, using a c5d.24xlarge instance and ubuntu 20.04 operating system (AMI ID: ami-03d5c68bab01f3496). This instance type has 96 logic cores, and 192 gigabytes of system memory. Input files are placed on a raid 0 logical volume created from the 4 NVME SSD devices available on this instance, with the following linux commands:

- mdadm --create /dev/md0 --level=stripe --raid-devices=4 /dev/nvme{1,2,3,4}n1
- mkfs -t ext4 /dev/md0

Because the linux operating system kernel caches all file read operations into unused system memory, the subsequent experiments reading the same files will not incur actual disk operations. To maintain the same conditions between experiments, the kernel caches are manually dropped using the following linux command between benchmark runs

- echo 3 > /proc/sys/vm/drop_caches

Note that this is different from disabling cache altogether. We permit caching during a single experiment run so that parallel tasks reading the same sections of a file (though rarely) can benefit from system cache.

Elapsed times are measured using the “gnu time” utility shipped with the operating system.

3.2. Benchmarking using Lustre distributed file storage on local compute clusters

We performed benchmarking experiments using our local high performance compute cluster hosted at the Center for High Performance Computing at the University of Utah. We have access to a 500 terabyte Lustre distributed file system as part of our multi-group shared storage. Lustre is a complex file system that has multiple layers of caching that is beyond the control of an end-user; and it serves multiple research groups and users. Therefore, the exact benchmarking conditions are difficult to reproduce. Our results represent our best effort at minimizing test environment variabilities.

The server that has access to the file system has 80 hyper-threaded cores and 384 gigabytes of system memory. We had exclusive access to this server during our benchmarking experiments. Local cache is cleared between experiments in the same way as described in ***method 3.1***.

4. Datasets

We used two datasets to conduct all our benchmarking experiments. The first is the publicly and openly available Genome In A Bottle (GIAB)[(Zook *et al.*, 2016)](https://paperpile.com/c/yAhGc9/ZGN9) Ashkenazim Trio Illumina 2x250bp GRCh38 BAM files. The inclusion of this dataset is to facilitate result reproduction. The second is a tumor-normal sample pair from a published study[(Huang *et al.*, 2021)](https://paperpile.com/c/yAhGc9/5XFt), Bn2 and Germ1 specifically, that is under controlled access. While gaining access to this dataset is more tedious, it serves to provide a more appropriate cancer content for tools like FACETS since tumor genomes can be highly aberrant.

5. Flagstats benchmark experiment

We ran the flagstats utility as part of samtools v1.16.1 for our benchmark experiments. We used the ‘-@’ parameter to control the number of threads samtools can create.

6. Snp-pileup benchmark experiment

We used the VCF recommended by the snp-pileup documentation (ftp://[ftp.ncbi.nlm.nih.gov/snp/organisms/human_9606/VCF/00-common_all.vcf.gz](http://ftp.ncbi.nlm.nih.gov/snp/organisms/human_9606/VCF/00-common_all.vcf.gz)) for sites of known polymorphism. For the stock version of snp-pileup, we used the ‘-d 2000’ option to limit the pileup depth, and the ‘-x’ option because the multiple pileup engine in quickBAM does not currently re-adjust base qualities of overlapping mates. We ran the stock snp-pileup with one thread, repeated three times to obtain standard deviations. No further runs were performed since the stock snp-pileup does not support multi-threading.

**DATA AVAILABILITY**

No new data were generated or analyzed in support of this research.

GIAB Ashkenazim Trio HG002 and HG004 Illumina 2x250bp novoalign GRCh38 BAM files are available at

- <https://ftp-trace.ncbi.nlm.nih.gov/ReferenceSamples/giab/data/AshkenazimTrio/HG002_NA24385_son/NIST_Illumina_2x250bps/novoalign_bams/>
- ​​<https://ftp-trace.ncbi.nlm.nih.gov/ReferenceSamples/giab/data/AshkenazimTrio/HG004_NA24143_mother/NIST_Illumina_2x250bps/novoalign_bams/>

The rapid autopsy tumor normal sample dataset was from a published study[(Huang *et al.*, 2021)](https://paperpile.com/c/yAhGc9/5XFt).

Known polymorphism sites VCF used for the snp-pileup benchmark experiments are available at

- <ftp://ftp.ncbi.nlm.nih.gov/snp/organisms/human_9606/VCF/00-common_all.vcf.gz>
